# Plasma membrane events associated with the meiotic divisions in the amphibian oocyte: insights into the evolution of insulin transduction systems and cell signaling

**DOI:** 10.1186/1471-213X-13-3

**Published:** 2013-01-23

**Authors:** Gene A Morrill, Adele B Kostellow, Richard D Moore, Raj K Gupta

**Affiliations:** 1 Department of Physiology and Biophysics, Albert Einstein College of Medicine, Bronx, New York 10461, USA; 2 The Biophysics Laboratory, State University of New York, Plattsburgh, NY, 12901, USA

**Keywords:** Insulin, Progesterone, Meiosis, Oocyte, Ouabain, Na/K-ATPase, Protease

## Abstract

**Background:**

Insulin and its plasma membrane receptor constitute an ancient response system critical to cell growth and differentiation. Studies using intact *Rana pipiens* oocytes have shown that insulin can act at receptors on the oocyte surface to initiate resumption of the first meiotic division. We have reexamined the insulin-induced cascade of electrical and ion transport-related plasma membrane events using both oocytes and intact plasma membranes in order to characterize the insulin receptor-steroid response system associated with the meiotic divisions.

**Results:**

[^125^I]Insulin binding (K_d_ = 54 ± 6 nM) at the oocyte plasma membrane activates membrane serine protease(s), followed by the loss of low affinity ouabain binding sites, with a concomitant 3–4 fold increase in high affinity ouabain binding sites. The changes in protease activity and ouabain binding are associated with increased Na^+^/Ca^2+^ exchange, increased endocytosis, decreased Na^+^ conductance resulting in membrane hyperpolarization, increased 2-deoxy-D-glucose uptake and a sustained elevation of intracellular pH (pH_i_). Hyperpolarization is largely due to Na^+^-channel inactivation and is the main driving force for glucose uptake by the oocyte via Na^+^/glucose cotransport. The Na^+^ sym- and antiporter systems are driven by the Na^+^ free energy gradient generated by Na^+^/K^+^-ATPase. Shifts in α and/or β Na^+^-pump subunits to caveolar (lipid raft) membrane regions may activate Na/K-ATPase and contribute to the Na^+^ free energy gradient and the increase in both Na^+^/glucose co-transport and pH_i_.

**Conclusions:**

Under physiological conditions, resumption of meiosis results from the concerted action of insulin and progesterone at the cell membrane. Insulin inactivates Na^+^ channels and mobilizes fully functional Na^+^-pumps, generating a Na^+^ free energy gradient which serves as the energy source for several membrane anti- and symporter systems.

## Background

The structure of insulin, its receptor and the post-receptor signaling pathways have been highly conserved during evolution. Both insulin and its receptor are similar in insects, mollusks and man [1,2]. As noted by Ebberink et al. [1], the various phyla have had a polyphyletic origin: i.e., the four major groups - the chordates and vertebrates, the echinoderms and tentaculates, the coelenterates, and the mollusks, worms, and arthropods - are now considered to have evolved independently of each other. Since insulin occurs in at least two main branches of the phylogenetic tree, it was probably present in the Archaemetazoa.

Insulin and insulin-like growth factors can act at the cell surface of the amphibian oocyte to initiate a cascade of events in preparation for cell division and differentiation (reviewed in [3,4]), although most studies use *X. laevis* ovarian follicles from females preinjected with progesterone. We have examined the role of insulin in the release of the prophase block in *R. pipiens* oocytes, which are excellent experimental material, both because of their large size (2 mm diameter), and the fact that each female contains 2–3 thousand oocytes that can be induced to undergo synchronous meiotic divisions *in vitro*. Intact oocytes can be stripped of all adhering follicle cells and plasma*-*vitelline membranes can be isolated [5] for ligand binding studies [6]. The prophase oocyte maintains a sizeable pool of high energy phosphate compounds, including phosphocreatine (PCr), ATP and serine-rich phosphoproteins, for at least 24 h during superfusion *in vitro.* Changes in cell surface area are monitored using voltage clamp techniques [7]. Superfusion of isolated oocytes or follicles in an NMR tube maintains physiological oxygen levels and allows analysis of changes in bioenergetics, intracellular cation levels and in membrane conductance (e.g. [8]).

We have examined the cascade of insulin-induced plasma membrane events in order to characterize both the insulin receptor and the membrane enzyme systems modulated by insulin during the first meiotic division. We find that when insulin binds to the plasma membrane receptor of the intact prophase *R. pipiens* oocyte, increased membrane serine protease activity and Ca^2+^ efflux are seen within minutes. This, in turn, initiates a transient increase in endocytosis (membrane recycling), resulting in a 3–4 fold increase in electrogenic Na^+^-pump sites, generating a large and sustained Na^+^ free energy gradient across the plasma membrane. This gradient is coupled to a number of secondary transporters that employ the downhill flow of Na^+^ to power the uphill flow of another ion and/or nutrient. (In antiporters (3Na^+^/Ca^2+^, Na^+^/H^+^) the chemical species move in opposite directions. In symporters (2Na^+^/glucose) the two species move in the same direction). Secondary transporters are ancient molecular machines, common in bacteria and archaea as well as in eukaryotes, and which probably preceded the insulin transduction system (reviewed in [9]). An increase in intracellular pH occurs over the next several hrs, followed by nuclear membrane breakdown by 8–10 h. Continuation of the meiotic divisions to second metaphase arrest requires the additional stimulus of progesterone and its polar metabolites.

## Results and discussion

### Comparison of mammalian and amphibian insulin and insulin receptor structures

**Figure 1:**
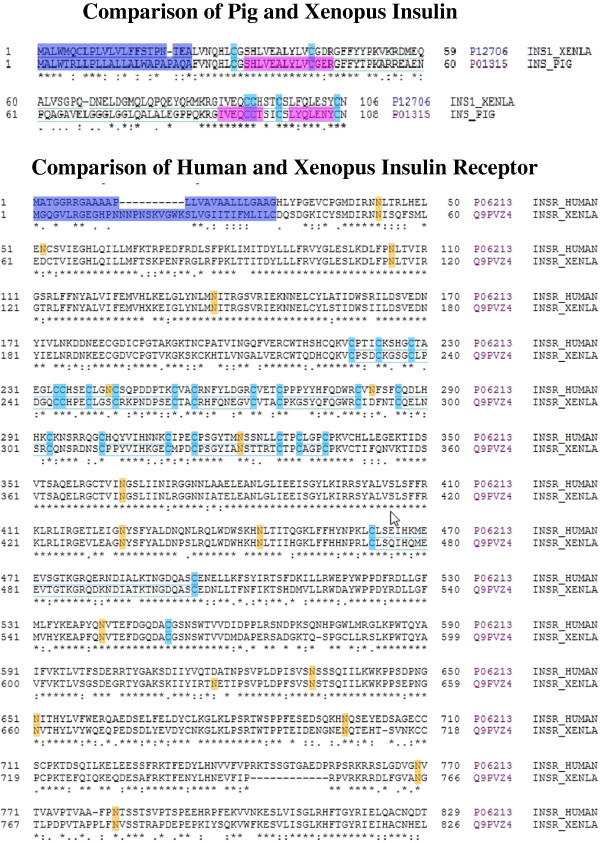
**Comparison of mammalian and *Xenopus laevis: *insulin and insulin receptor structures.** The upper panel compares the amino acid sequences of Porcine (Accession #P01315) and *X. laevis* (Accession #P12706) insulin-1. The lower panel compares the alignment of the α-subunits of the *X. laevis *insulin receptor (Accession #Q9PVZ4; sequence 38–754) and human insulin receptor (Accession #P06213; sequence 28–758). Data are from the unprocessed sequences as listed in the Swiss Protein Knowledgebase (http://www.uniprot.org).

The upper panel of Figure 1 compares the amino acid sequences of Porcine (Accession #P01315) and *Xenopus laevis* (Accession #P12706) insulin-1. Porcine insulin (Eli Lilly and Company, see methods) is frequently used in biological research and is compared here with peptides native to frogs. Using *X. laevis* insulin-1 as an example of frog insulin, the unprocessed insulin sequence as listed in the Protein Data Base (http://www.uniprot.org) includes a putative signal peptide (1–23), the insulin B-chain (24–53), C peptide (56–83) and the insulin A-chain (86–106). The insulin precursor is further processed into a oligotetramer containing two alpha and two beta subunits [10]. The align function of the Protein Knowledgebase compares the amino acids that are associated with the signal peptides (violet), disulfide bridges (blue) and glycosylation sites (orange). The regions in red indicate helices. The symbol (*) below each amino acid pair in Figure 1 indicates a commonality at that position whereas (:) indicates structurally related amino acids.

The lower panel of Figure 1 compares the alignment of the α-subunits of the *X. laevis* insulin receptor (Accession #Q9PV24; sequence 38–754) with the human insulin receptor (Accession #P06213; sequence 28–758). (The amino acid sequence for the pig insulin receptor is not available.) The insulin receptor sequence depicts the α-subunit represented by the N-terminal 826 and 829 amino acids of *X. laevis* and human insulin, respectively. The α-subunit of the insulin receptor contains the insulin binding site(s) (reviewed in [11]). The C-terminal sequence of the unprocessed insulin receptor containing the β-subunit is not shown. In general, based on the position of disulfide bridges, glycosylation sites and amino acid sequence, the structures of both the unprocessed insulin chains and the α-subunit of the insulin receptor have been highly conserved from frog to mammals.

### Relative contributions of insulin and progesterone to the release of the prophase block

In vertebrates, the ovarian oocyte is blocked in first meiotic prophase and remains so until gonadotropin release prior to ovulation. In amphibians, gonadotropins stimulate oocyte surface epithelial cells to synthesize progesterone [12]. Progesterone, diffusing from the follicle cells, binds to the α-subunit of the Na/K-ATPase [13] to release the prophase block. In the frog ovary, meiosis then continues to 2nd meiotic metaphase and the egg undergoes ovulation. Fertilization releases the metaphase block and meiosis is completed within 15–20 minutes followed by the mitotic divisions which form the developing blastulae (reviewed in [14]). In *R. pipiens*, release of the prophase block is associated with a swelling of the large germinal vesicle or nucleus after 6–8 h at 20°C *in-vitro*. The nucleus rises to the animal pole and pushes aside the black cortical pigment granules, forming a white depigmented region often used as an index for “meiotic maturation”. At 2nd metaphase arrest the first polar body appears as a black spot within the whitish area at the animal pole after 12–15 h (see [15]).

Table 1 compares the effect of insulin and progesterone on meiosis in *R. pipiens* oocytes free of follicle cells (denuded). If taken from hibernating females in winter (December - January), insulin alone does not induce nuclear membrane breakdown, but by early spring, insulin produces breakdown in about 50–75% of the oocytes, although none continue to 2nd metaphase arrest. For comparison, progesterone alone induced more than 95% of the denuded oocytes to undergo nuclear membrane breakdown in winter and spring; the dose required decreased 100-fold with approaching spring [16]. Only about 30% of the progesterone-treated oocytes reached 2nd metaphase arrest. As shown in Table 1, combining insulin and progesterone produced a maximal (>99%) meiotic response by spring. As reported elsewhere, further metabolism of progesterone to one or more polar steroids is necessary for nuclear membrane breakdown [6]. Polar steroids alone do not induce nuclear membrane breakdown [5].

**Table 1:** **Comparison of porcine insulin and progesterone on the meiotic response using denuded *Rana pipiens *Oocytes**

**Additions**^**1**^	**Meiotic response**	**Meiotic response**
	**% Nuclear breakdown**	**% 2nd Metaphase arrest**
None	0 (6)^2^	0 (6)
Insulin, 10 μM	74 ± 5.2 (4)	0 (4)
Progesterone, 0.32 μM	97 ± 3.8 (4)	29 ± 3.8 (4)
Insulin , 10 μM + Progesterone, 0.32μM	99 ± 2 (4)	96 ± 1.1 (4)

^1^Incubated in modified Ringer’s solution for 24 h at 20–22ºC. Experiments were carried out in February-March. Values shown are the maximal response.

^2^Mean ± SD (Number of frogs).

Table 1 thus indicates that both insulin and progesterone are essential for completion of the first one and one-half meiotic divisions leading to production of a fertilizable egg. Based on these and other studies (e.g. [16]) the response system in *R. pipiens* appears to be activated in late spring by release of both insulin and progesterone as the female comes out of hibernation. Insulin was first reported to induce nuclear membrane breakdown in isolated *X. laevis* oocytes in which follicle cells were usually removed by protease treatment [17-20]. Insulin-like growth factor (IGF-1) is reported to be 60–100 times more potent than insulin in stimulating meiosis although both insulin and IGF-1 are present in the frog (reviewed in [21]). However, the *X. laevis* ovary contains follicles in different growth stages and may be acting on oocytes still undergoing growth. Furthermore, interpretation of the findings is complicated by the fact that *X. laevis* females were generally injected with gonadotropin 3–5 days before excising the ovaries. Since gonadotropin stimulates progesterone synthesis by amphibian ovarian follicle cells [12], some of the *X. laevis* studies may have involved an insulin response in progesterone-primed ovarian follicles still in the growth phase.

### [^125^I]Insulin binding to the oocyte plasma membrane

The vertebrate insulin receptor is thought to bind two insulin molecules on each of two α-subunits (reviewed in [22]). We have incubated follicle cell free (denuded) *R. pipiens* oocytes in Ringer’s solution containing ^125^I]insulin for varying times under physiological conditions, quickly rinsed and removed the plasma-vitelline membranes intact (see [5]), and measured ^125^I] in membranes, cytosol and intact oocytes. We previously reported that maximal ^125^I]insulin binding to the oocyte plasma-vitelline membrane complex occurs within 15 minutes at 20–22°C, although ^125^I]insulin continues to be taken up into the cytoplasm for at least 2 h [6]. An additional constant binding ^125^I] membrane fraction [6] largely disappeared following transfer of the oocytes to medium containing unlabeled insulin for 15–30 min. The ^125^I]insulin displaced by unlabeled insulin was recovered in the oocyte cytosol/particulate fractions.

**Figure 2:**
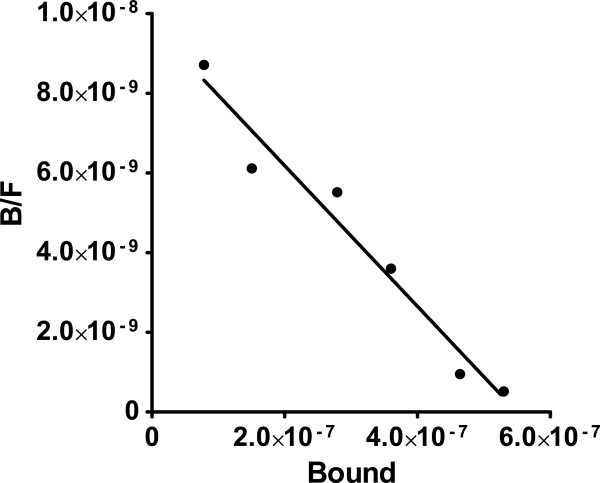
**Scatchard type plot of [**^**125**^**I]insulin binding to the plasma-vitelline membrane complex of denuded *R. pipiens *oocytes.** Intact, denuded prophase oocytes were incubated in Ringer’s solution containing [^125^I]insulin for 15 min at 20–22°C, the oocytes removed, rinsed with Ringer’s solution and the membranes removed and counted as described in Methods. The [^125^I]insulin binding data were corrected by subtracting the constant binding fraction.

Scatchard-type analysis (Figure 2) of the prophase *R. pipiens* oocyte (after subtracting the constant binding fraction) indicates a single high affinity [^125^I]insulin binding site with K_d_ = 53.4 ± 6.3 nM and a binding capacity of 0.54 ± 0.062 fmols/oocyte. Assuming a 2:1 relationship between insulin and membrane receptor sites, the x-intercept indicates about 2.8 × 10^6^ sites per oocyte. For an oocyte 1.8 mm in diameter, there would be a maximum of about 30 insulin-binding sites per μm^2^. No measurable Scatchard type [^125^I]insulin binding to plasma-vitelline membranes was found when oocytes were pretreated with inducing levels (1.65 μM) of progesterone for 30 min, suggesting that the insulin receptor is internalized following progesterone binding to the Na^+^/K^+^-ATPase α-subunit.

Diss and Greenstein [23] reported that ^125^I]insulin displayed sigmoidal binding behavior with X. laevis Stage V/VI oocytes after uptake for 1 h at 15° C. They resolved the resulting curvilinear Scatchard type plot into two components with K_d_ values of 8.86×10^-10^ M and 5.32×10^-9^ M. The different K_d_’s may reflect the fact that intact *X. laevis* oocytes with follicle cells and vitelline membranes removed were used for binding studies. The possible contribution of non-specific endosome/cytosol sequestration was not mentioned.

### Sequential insulin-induced changes in plasma membrane transport/exchange systems in intact, denuded *R. pipiens* oocytes

**Figure 3:**
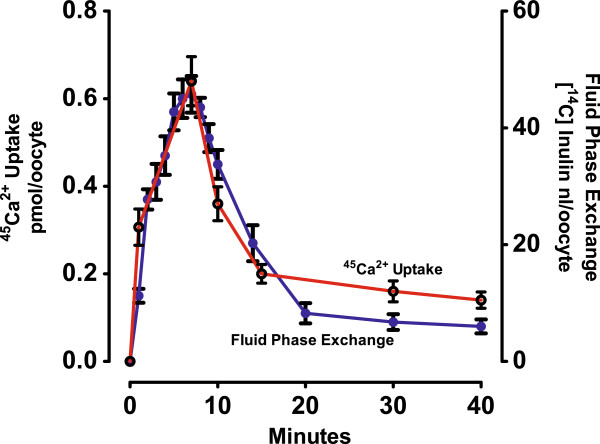
**A comparison of the time course of insulin effects on **^**45**^**Ca**^**2+**^**/Na**^**+ **^**exchange and fluid phase uptake ([**^**14**^**C]insulin) by isolated denuded *R. pipiens *oocytes at 20–22°C, plotted as rate of change per unit time (dy/dt). **The values are in pmols/oocyte for ^45^Ca^2+^ and [^14^C]inulin, respectively. Each point presents a pooled sample of 5–10 sibling oocytes and the values shown are typical for 4 experiments in late winter-early spring. For details see Methods.

Figure 3 compares the time course of two transient insulin-induced early membrane-associated events: 1) fluid phase exchange (endocytosis: measured by ^14^C]inulin uptake [24]) and 2) ^45^Ca^2+^ uptake and exchange [25]. Values are expressed as rate of change (dy/dt), relative to controls in intact denuded sibling oocytes incubated in normal (114 mM Na^+^) amphibian Ringer’s solution (see Methods). Inulin, a 5000–5500 molecular weight polysaccharide, is in general not enzymatically degraded or actively transported by cells and is used to measure fluid phase turnover (endocytosis) in isolated cell systems (see [24]). The simultaneous transient increase in both ^14^C]inulin and ^45^Ca^2+^ uptake during the first 10 minutes (Figure 3) indicates an initial insulin-induced increase in plasma membrane recycling.

Comparison of ^14^C]inulin uptake into the oocyte cytosol and isolated plasma-vitelline membranes indicates that 15–20% of the total ^14^C]inulin taken up during the first 15–30 min was associated with a constant binding fraction in the plasma-vitelline membrane [24]. Pulse-chase experiments followed by extraction and chromatography of the cytosol and constant binding fractions demonstrated that ^14^C]inulin was internalized but not bound or metabolized. Comparison of membrane-sequestered ^125^I]insulin with that of ^14^C]inulin, suggests that the membrane constant binding fraction is contained within the numerous endocytotic vesicles seen in electron micrographs of the fully grown prophase oocyte [7,8].

**Figure 4:**
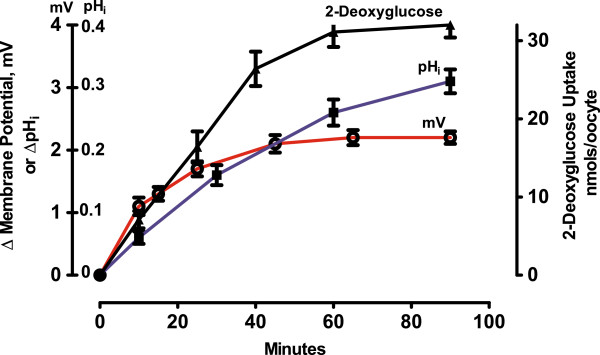
**A comparison of the time course of insulin effects on membrane hyperpolarization (E**_**mV**_**), intracellular pH (pH**_**i**_**) and [**^**14**^**C]2-deoxy-D-glucose uptake by isolated denuded *R. pipiens *oocytes at 20–22°C. **The graphs represent changes in various parameters relative to untreated oocytes. Each point presents a pooled sample of 5–10 sibling oocytes and is typical for 4 experiments in late winter-early spring *Rana*. For details see Table 2 and Methods.

Figure 4 indicates that activation of three plasma membrane transport systems immediately follow the early ^45^Ca^2+^ and ^14^C]inulin transients, causing 1) membrane hyperpolarization (E_mV_), 2) ^14^C]2-deoxy-D-glucose uptake, and 3) increase in intracellular pH (pH_i_). Membrane hyperpolarization begans within 5–10 min followed by an increase in 2-deoxyglucose uptake and increase in intracellular pH. Both glucose uptake and membrane hyperpolariztion approached a new steady-state by 40–60 min. Intracellular pH approached a maximal value after about 3 h (data not shown). The increase in intracellular pH and associated increase in ^22^Na^+^ influx are blocked by amiloride, indicating that insulin stimulates an amiloride-sensitive Na^+^/H^+^ exchange system [6,25].

Based on the specific activity of the medium and the inulin uptake into the cytoplasm, it is estimated that a fluid volume of 20–25 nl is internalized per oocyte per hour [24]. At a steady-state, this fluid exchange accounts for about 40% of intracellular Na^+^ turnover in the unstimulated (control) oocyte [24]. The observed 2–3 fold insulin-induced increase in fluid phase uptake (endocytosis) and Na^+^/Ca^2+^ exchange increased intracellular Na^+^ concentration within the first 15–20 min (data not shown).

### Insulin-induced activation of plasma membrane serine protease

**Figure 5:**
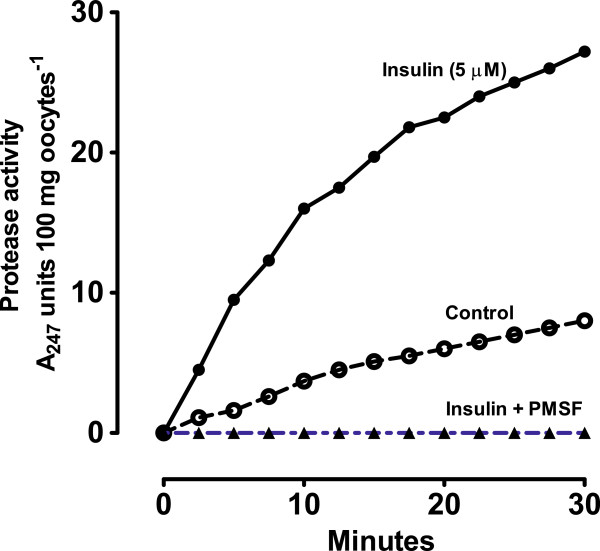
**Effect of 5 μM insulin on serine protease activity in isolated, intact plasma-vitelline membranes from prophase-arrested *Rana pipiens *oocytes (● - ●). **p-Tosyl-l-arginine methyl ester (TAME) was used as a serine protease-specific substrate and the serine protease inhibitor phenylmethyl-sulfonyl-flouride (PMSF) was added 15 min prior to insulin (▲ - ▲). The control is represented by (○ - ○). Values shown are from a typical experiment using sibling oocytes. The mean ± SD values for serine protease activity for oocytes from four *R. pipiens* females at 30 min was 26.4 ± 2.57 absorbancy units for 5 μM insulin, 8.23 ± 1.27 absorbancy units for 0 insulin (controls) and 0 absorbancy units for oocytes treated with both 5 μM insulin and PMSF. For details see methods.

Figure 5 demonstrates that insulin rapidly activates a serine protease in plasma membranes isolated from prophase *R. pipiens* oocytes. As also shown, the serine protease inhibitor phenylmethylsulfonyl fluoride (PMSF) inhibited insulin activation of membrane protease. PMSF binds specifically to the active site serine in a serine protease [26]. It does not bind to any other serine residue in the protein. Inhibition of insulin-induced protease activation occurred only if protease inhibitors (PMSF, leupeptin, etc.) were added prior to insulin. No effect was seen if the inhibitor was added at the same time as insulin, and the inhibitors alone had minimal effect on oocyte membrane potential, phosphocreatine levels, and intracellular pH.

Seals and Czech [27] were the first to demonstrate that binding of insulin to plasma membranes (adipocytes) activates membrane protease. Four protease-activated receptors (PARs) have now been identified, one of which (PAR2) is activated by serine proteases. PARs are G-protein-coupled receptors, activated by cleaving an N-terminal sequence (reviewed in [28]). The mechanisms that control PAR2 signaling are not well defined, but once activated, PAR2 undergoes internalization and sorting [28] which may contribute to the transient increase in membrane recycling shown in Figure 3. It is interesting to note that endogenous serine proteases induce meiosis in oocytes of the annelid *Sabellaria* and that serine protease inhibitors block meiosis in *Xenopus* and *Rana* oocytes (reviewed in [29]). This suggests that protease activation was an early evolutionary step [30] in meiotic induction and that insulin may have subsequently evolved as a mechanism for fine-tuning release of the prophase block.

### Insulin-induced electrical and ionic changes at the oocyte plasma membrane

Table 2 summarizes both insulin- and progesterone-dependent changes in: 1) fluid phase exchange (nl/oocyte), 2) plasma membrane potential (E_mV_), 3) plasma membrane conductance (μS), 4) 2-deoxyglucose uptake (nmols/h/oocyte) and 5) intracellular pH (pH_i_) during incubation of prophase oocytes. Insulin and progesterone-dependent changes are compared in physiological (114 mM Na^+^) Ringer’s solution and Na^+^-free Ringer’s solution. The bottom line of Table 2 indicates that preincubation with the serine protease inhibitor, PMSF, blocks all insulin induced membrane events studied: endocytosis, hyperpolarization, increase in pH_i_, and 2-deoxyglucose uptake. Of the insulin-induced membrane responses shown, only one, increased pH_i_ , also occurred in response to progesterone. Another response, membrane conductance, decreased with insulin stimulation, whereas it increased with progesterone stimulation.

**Table 2:** **Insulin-induced time-dependent changes in intact denuded *Rana pipiens *Oocytes**^**1**^

**Additions**		**Fluid phase exchange**	**Membrane potential**	**Membrane conductance**	**2-Deoxy-glucose uptake**	**pH**_**i**_	**Na**^**+**^
		**nl/h/oocyte**	**mV**	**μS**	**nmols/h/oocyte**		**Electrode**^**2**^
None	+Na^+^	23.4 ± 1.23 (5)	−42.6 ± 1.6 (8)	5.2 ± 0.3 (14)	1.05 ± 0.19(3)	7.38 ± 0.035 (7)	44.2 ± 2.4 (3)
	-Na^+^	7.68 ± 1.8 (3)	−75.0 ± 4.2 (4)		0.12 ± 0.07 (3)	7.24 ± 0.040 (3)	
Insulin, 10 μM	+Na^+^	65.5 ± 2.4 (3)	−59.2 ± 1.7 (6)	1.8 ± 0.4 (4)	3.05 ± 0.29 (3)	7.65 ± 0.017 (3)	19.0 ± 2.0 (3)
	-Na^+^	7.94 ± 0.65 (3)	−69.0 ± 3.2 (4)		0.17 ± 0.06 (3)	7.26 ± 0.021 (3)	
Progesterone +Na^+^,1.65 μM		18.8 ± 0.29 (3)	−52.0 ± 4.5 (4)	6.4 ± 0.5 (8)	1.03 ± 0.16 (3)	7.69 ± 0.34 (4)	**........**
Insulin, 10 μM + Na^+^ +PMSF, 1.0 mM^3^		13.8 ± 1.7 (3)	−38.0 ± 1.5 (6)	5.0 ± 0.4 (4)	1.11 ± 0.23 (3)	7.24 ± 0.26 (3)	**........**

^1^All values are means ± SD (N = number of frogs); 60–90 min incubation period in 114 mM Na^+^ or Na^+^-free Ringer’s solution at 20–22°C.

^2^Na^+^-selective microelectrode [66]. Measured 1 h after exposure to Ringer’s solution containing 10 μM insulin.

^3^15 min pretreatment with 1.0 mM PMSF (see Methods).

Extracellular Na^+^ is essential for insulin stimulation of endocytosis (column 2), 2-deoxyglucose uptake (column 5) and increase in pH_i_ (column 6). Endocytosis increases 2–3 fold during the first 20 minutes after exposure to insulin and largely accounts for the initial increase in intracellular Na^+^. An estimate of both membrane surface area (based on membrane capacitance measurements) and endocytotic vesicle size (from electron micrographs) [7] indicates that, on average, the oocyte plasma membrane recycles several times an hour. The data in columns 3 and 4 of Table 2 indicate that membrane hyperpolarization is Na^+^-dependent and that the negative-going hyperpolarization (Figure 4) is largely due to a marked decrease in Na^+^ conductance. Hyperpolarization generally began within 10 min: the potential usually reaches a new steady state within 60–90 min and the oocyte remains hyperpolarized for 4–5 h. In oocytes from 8 females, the insulin-induced hyperpolarization was 17 ± 2 mV. These values are typical for denuded oocytes from November through March. By late spring, the resting potential of the denuded prophase oocyte increased to 70–80 mV (inside negative) and addition of insulin no longer produced hyperpolarization.

The hyperpolarizing effect of insulin is compared in the presence of a Na^+^-channel blocker (amiloride), a Na^+^ pump inhibitor (strophanthidin) and a serine protease inhibitor (PMSF) in Table 3. Amiloride mimicked the low Na^+^ medium, producing a 26 mV hyperpolarization that was insensitive to insulin. As also shown, addition of insulin to strophthantidin-treated oocytes produced a 20 mV hyperpolarization. Treatment of voltage-clamped prophase oocytes with 10 μM strophanthidin resulted in a rapid (less than 1 min) decrease in membrane current without a significant change in membrane conductance (data not shown). The decrease in conductance in response to insulin is not due to hyperpolarization, per se, since the steady-state conductance of the untreated oocyte increases when voltage-clamped above its resting potential. Pretreatment with the serine protease inhibitor PMSF had minimal effect on resting membrane potential but did block insulin-induced hyperpolarization.

**Table 3:** **Effect of insulin on the plasma membrane potential of the denuded rana oocyte as a function of external Na**^**+ **^**and inhibitors of Na**^**+ **^**channels/Na**^**+**^**:H**^**+ **^**exchange, Na**^**+**^**/K**^**+**^**-ATPase and serine protease activity**

**Medium**	**E**_**mV **_**control**	**E**_**mV **_**10 μM insulin**^**a**^
Ringer’s Solution (114 mM Na^+^)	−42.6 ± 1.6 (8)^b^	−59.2 ± 1.7 (8)
2.5 mM Na^+^-Ringer’s Solution	−75.0 ± 1.0 (10)	−69.0 ± 3.0 (5)
1.0 mM Amiloride/Ringer’s Solution	−68.7 ± 1.4 (7)	−69.0 ± 2.1 (5)
10 μM Strophanthidin/Ringer’s Solution	−26.4 ± 1.0 (10)	−45.7 ± 0.6 (10)^c^
1 h pretreatment with 1.0 mM PMSF in Ringer’s Solution	−39.1 ± 1.5 (7)	−38.0 ± 1.5 (12)
1.0 mM PMSF added with insulin		−60.1 ± 3.2 (7)

^a^Mean ± SE 60–90 min after addition of insulin. “Instantaneous” conductance was 3.2 ± 0.5, 1.85 ± 0.4 and 1.30 ± 0.3 μS for untreated, insulin-treated and amiloride-treated oocytes, respectively.

^b^ Number of sibling oocytes.^c^30-60 min after addition of 10 μM strophanthidin

From the data shown in Tables 2 and 3, oocyte membrane conductance appears largely associated with Na^+^ channels. Na^+^ channels are classified according to the trigger that opens the channel, i.e. either “voltage-gated” or “ligand-gated” [30,31]. As shown in Table 3, oocyte Na^+^ conductance is amiloride sensitive, indicating a non-voltage-sensitive ion channel belonging to the amiloride-sensitive Na^+^ channel (TC 1.A.6) family. Regulation of epithelial Na^+^ channel function seems to be achieved through cell surface insertion/retrieval of channels, by changes in channel open probability, or through a combination of these processes (reviewed in [28]). The large α-subunit of the channel associates with one or more accessory subunits (β1, β2 and β3) and channel activity is modulated by multiple factors, including proteases (reviewed in [28,31,32]). The finding that insulin activates oocyte serine protease (Figure 5) and that a serine protease inhibitor (PMSF) blocks insulin-induced membrane hyperpolarization (row 5, Table 2) suggests that activation of a serine protease(s) is responsible for Na^+^ channel inactivation and resulting membrane hyperpolarization.

2-Deoxyglucose uptake is also Na^+^-dependent (Table 2). Increased uptake follows the transient rise in endocytosis (membrane recycling) and correlates with membrane hyperpolarization (Figure 4). Na^+^-dependent glucose transporters, SGLTs, are a broad class of integral membrane proteins that mediate thermodynamically-coupled transport of sugars and Na^+^ (reviewed in [33]). Vera and Rosen found [34] that the endogenous *X. laevis* oocyte glucose transporter was kinetically and immunologically indistinguishable from both brain and liver glucose transporters, known to be insulin responsive. They also found that upon expression, two different insulin-insensitive mammalian glucose transporters became insulin-responsive in the oocyte. This suggests that insulin-sensitivity (and possibly Na^+^-dependence) is cell-dependent.

### The oocyte membrane Na^+^/K^+^-ATPase and Na^+^ channels modulate the Na^+^ free energy gradient

The insulin-induced decreased Na^+^ channel conductance, coupled with a decrease in cytoplasmic free Na^+^ ions (due in part to stimulation of plasma membrane Na^+^/K^+^-ATPase) generates a Na^+^ electrochemical potential difference (gradient) that can be used as an energy source for the activation of Na^+^/glucose symporter, as well as the 3Na^+^/Ca^2+^ (seen as Ca^2+^ efflux) and Na^+^/H^+^ (seen as increased pH_i_) antiporters. Insulin usually stimulates the co-transport of 2 Na^+^ ions for each glucose molecule. The ability of SGLT’s to accumulate glucose is critically dependent on the stoichiometry of the Na^+^ and glucose fluxes, i.e. [Glucose]_i_/[Glucose]_o_ = ([Na^+^]_o_/[Na^+^]_i_)^n^/exp(nV_m_F/RT), where i and o are intracellular and extracellular glucose and Na^+^, respectively, V_m_ is the membrane potential (inside negative, expressed as a negative number), F is the Faraday constant, R is the gas constant, T is the absolute temperature, and n is the coupling coefficient. The V_m_ and [Na^+^]_i_ values in Table 2 indicate that, in oocytes, insulin would stimulate a 20-fold increase in glucose uptake. Similar calculations can be made for H^+^ and Ca^2+^ ions and would explain increased Ca^2+^ and H^+^ efflux. Thus, the insulin effects reported here appear largely due to an increased Na^+^-free energy gradient.

Moore first reported that insulin stimulates a Na^+^ and amiloride-sensitive increase in intracellular pH in frog skeletal muscle *in vitro*[35]. Subsequent studies by Vigne et al. [36] failed to confirm the insulin-induced increase in intracellular pH, using myoblasts from 9–12 day old chick embryos. However, Vigne et al. incubated myoblasts in medium containing 25 mM Hepes-Tris at pH 7.4. Passive diffusion and/or fluid phase exchange (endocytosis) may have facilitated uptake of Hepes-Tris buffer into the myocytes and the increased buffering capacity could have prevented the physiological insulin-induced intracellular pH changes. More recently, Yang et al. [37] found that insulin treatment of cadiomyocytes resulted in a marked alkalinization of the cytoplasm as measured using a fluorescent indicator, confirming the earlier findings. Yang et al. [37] further found that the alkalinizing effect of insulin was blocked with cariporide, consistent with stimulation of Na^+^/H^+^ exchange.

### Effect of insulin on ouabain-binding to the oocyte plasma-vitelline membrane

Table 4 compares ^3^H]ouabain binding to denuded oocytes with and without 10 μM insulin for 60 min. Isolated denuded oocytes were then incubated with ^3^H]ouabain and the intact plasma-vitelline membrane complex isolated and counted as described in Methods. (^3^H]ouabain binding to the oocyte plasma-vitelline membrane was maximal within 60 min, but ouabain continued to be taken up into the cytoplasm for several hours [6].) Based on the Scatchard plot for ouabain binding, there are 40–50 Na^+^-pump sites per insulin binding site, and in the untreated oocyte, the low affinity ouabain binding sites predominate. In contrast, the insulin-treated oocyte displays a single class of high-affinity ouabain-binding sites and the density of high affinity sites has increased 3-4 fold. This is consistent with studies using human embryonic kidney (HEK-293) cells [38] in which insulin stimulated an increase in surface Na^+^-pump α-subunits. The increase in Na^+^-pump α-subunits correlated with an increase in the binding of an antibody that recognizes only the nonphosphorylated form of the α-1 subunit (at serine-18).

**Table 4:** **Effect of inducing levels of porcine insulin on [**^**3**^**H] Ouabain binding to the plasma-vitelline membrane of the *Rana Pipiens *oocyte in meiotic prophase arrest**^**1**^

**Additions**	**[**^**3**^**H] Ouabain binding K**_**d**_^**2**^	**Ouabain binding capacity fmols/oocyte**
None	2.0 ± 0.19 × 10^-6^ M	80 ± 4
	3.2 ± 0.12 × 10^-8^ M	15 ± 2
Insulin, 10 μM	4.9 ± 0.23 × 10^-8^ M	53 ± 3.3

^1^Means ± SD (N = 3 frogs).

^2^In the absence of insulin, ouabain binding was biphasic, with high and low affinity sites. In the presence of insulin the low affinity sites disappeared and the binding capacity of high affinity sites increased.

The question therefore arises, what is the nature of the high and low affinity ouabain binding sites? Using Fourier Transform infrared difference spectroscopy, Stotz et al., [39] have found that elevated Na^+^ concentrations prevent high affinity ouabain binding to purified porcine kidney Na/K-ATPase. Based on spectrofluorimetry and calorimetric titrations, they propose that when intracellular Na^+^ is elevated, low affinity ouabain binding is induced by binding of a third Na^+^ ion to Na^+^/K^+^-ATPase. They concluded that the cardiac glycoside binds preferentially to the phosphorylated state of the enzyme, and that only conformations with 0 or 2 bound Na^+^ ions permit high-affinity ouabain binding. Subsequently, Sandtner et al. [40] found that in *X. laevis* oocytes, the two ouabain sites are mutually exclusive, with the low affinity site at the cell surface near the ion permeation pathway [41] and the high affinity site deeper within the permeation pathway. The ouabain at the low affinity site has a similar position to that observed by X-ray crystallography [42]; however, the high affinity site is situated deeper within the channel and the lactone ring at C-17 faces outwards rather than inwards as indicated by the crystallography data.

These results indicate that ouabain traverses the permeation pathway from the low affinity to the high affinity site. If Stotz et al. [39] and Sandtner et al. [40] are correct, the high extracellular Na^+^ level at the membrane surface is responsible for maintaining low affinity ouabain binding and that a third Na^+^ is displaced as the ouabain moves down the permeation pathway. We found that insulin causes the disappearance of the low affinity ouabain binding within minutes, suggesting that insulin binding to its receptor results in a conformational change that prevents binding by a third Na^+^ ion and that optimizes formation of the energy transducing ion pump.

### Topology of the plasma membrane protease-PAR2-Na^+^ channel transduction system

**Figure 6:**
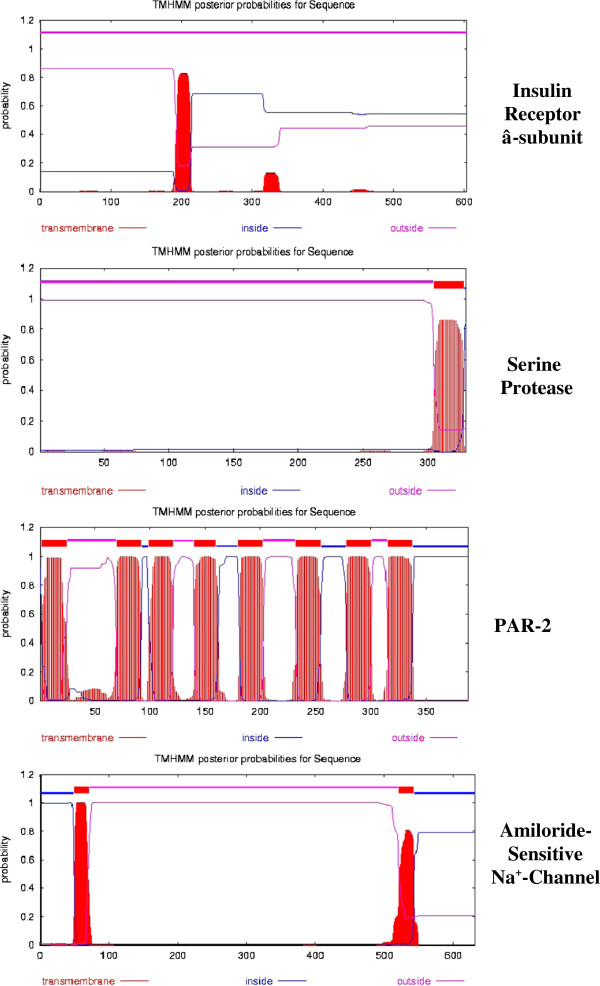
**A comparison of the topology of four plasma membrane proteins associated with the initial insulin response. **TMHMM projections were generated using the server at the Center for Bological Sequence Analysis, Technical University of Denmark DTU. From top to bottom the proteins are: the β-subunit of the insulin receptor (Accession #Q9PVZ4), the membrane serine protease 8 (Prostasin, Accession #Q16651), the protease-activated receptor (PAR2, Accession #Q5U791) and the amiloride-sensitive Na^+ ^channel subunit β (Accession #P51169). The amino acid sequences are those published in the Swiss Protein Knowledgebase (http://www.uniprot.org).

Figure 6 compares the topology of four putative plasma membrane peptides that are associated with the insulin-response system of the oocyte plasma membrane: 1) the β-subunit of the insulin receptor (Accession #Q9PVZ4), 2) the protease-activated receptor-2 (PAR2, Accession #Q5U791), 3) the membrane channel regulating serine protease (Accession #O42272) and 4) the amiloride-sensitive sodium channel subunit α (Accession #P37088). The vertical red bars indicate positions of the transmembrane (TM) helices.

The insulin receptor is composed of two α and two β subunits [3,4]; the β being inserted into the plasma membrane via a single transmembrane helix. Each α-subunit (not shown) binds two insulin molecules and is, in turn, attached to a β-subunit via S-S bridges. The TMHMM server predicts that each β-subunit of the insulin receptor and the serine protease has a single transmembrane (TM) helix whereas the protease activated receptor (PAR2) appears to have eight TM helices. A fourth response element, the amiloride-sensitive Na^+^-channel (bottom projection), exhibits 2 TM helices, located near the N- and C-terminal ends of the peptide.

In contrast to the TMHMM prediction, MEMSAT-3 analysis [43] of PAR2 predicts a 20 amino acid N-terminal signal peptide and seven TM helices. The secretory signal peptide is a ubiquitous protein-sorting signal that targets its passenger protein for translocation across the endoplasmic reticulum membrane in eukaryotes. Many methods have been published for predicting signal peptides from the amino acid sequence (see [44]), but most have a limited ability to distinguish between signal peptides and N-terminal TM helices (both are hydrophobic). Version 4.0 of SignalP, MEMSAT-3/SVM and Phobius attempt to solve this problem. Of these methods SignalP v. 4.0 appears to be the most consistent. SignalP v. 4.0 (see Methods) indicates the presence of a signal peptide cleavage site between positions 20 and 21. Thus, in agreement with MEMSAT-3/SVM, the first putative TM helix is a signal peptide, indicating that PAR2 contains only seven TM helices.

### Membrane topology of insulin-stimulated transporters in the oocyte plasma membrane transduction system

**Figure 7:**
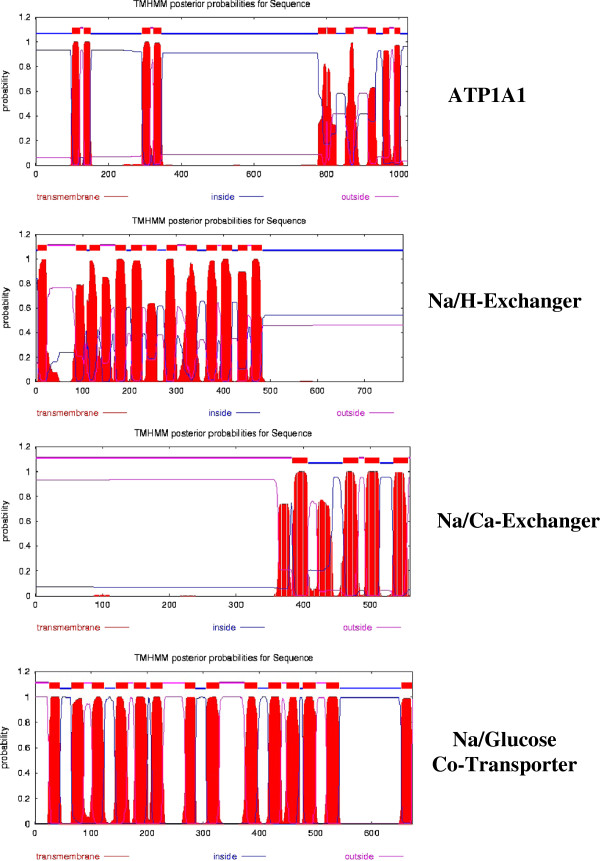
**A comparison of the topology of four putative plasma membrane enzymes that respond to exogenous insulin. **TMHMM projections were generated as described in Figure 6. From top to bottom the enzymes are: the catalytic α-subunit of Na^+^/K^+^-ATPase (Accession #Q92123), Na^+^/H^+^-exchanger (Accession #P70009), the Na^+^/Ca^2+^-exchanger (Accession #Q91849) and Na^+^ -glucose cotransporter ((SGLT2, Accession #P31639). The amino acid sequences are those published in the Swiss Protein Knowledgebase (http://www.uniprot.org).

Figure 7 compares the topology of three antiporters and one symporter associated with the insulin response in oocytes. The amino acid sequences are those published in the Swiss Protein Database (http://www.uniprot.org), and, with the exception of SGLT2, are those published for a closely related frog, *X. laevis.* SGLT2 has not been identified in *Xenopus*, therefore the topology for the human enzyme is shown. From top to bottom the enzymes are: the catalytic α-subunit of Na^+^/K^+^-ATPase (Accession #Q92123), the Na^+^/H^+^-exchanger (Accession #P70009), the Na^+^/Ca^2+^-exchanger (Accession #F7A1F8) and the insulin-regulated glucose-Na^+^ co-transport enzyme (SGLT2, Accession #P31639).

All 4 membrane enzymes are involved in Na^+^ exchange or in co-transport systems. Na^+^/K^+^-ATPase actively exchanges 3 Na^+^ for 2 K^+^, contributing to membrane hyperpolarization. The Na^+^/H^+^ antiporter exchanges 1 Na^+^ for each H^+^ expelled, resulting in an increase in intracellular pH. Similarly, the Na^+^/Ca^2+^ exchanger catalyzes a 3:1 exchange, often resulting in a small membrane depolarization. Finally, the Na^+^/glucose co-transporters take up 2 Na^+^ into the cell for every glucose molecule taken up. As in Figure 6, the vertical red bars indicate the positions of transmembrane (TM) helices. All four enzymes have multiple TM domains, often in groups or pairs. According to MEMSAT-SVM, the Na^+^/Ca^2+^ exchanger has 6 TM helices. From top to bottom, the four enzymes exhibit 10, 12, 6 and 14 TM helices, respectively.

Table 5 indicates that, in addition to multiple TM helices, the enzymes shown in Figures 6 and 7 contain 1 to 3 caveolin binding motifs [45]. Caveolins are a family of 22 kDa integral membrane proteins involved in receptor-mediated endocytosis. Systematic evolutionary analysis shows a conservation of genes encoding three caveolins in metazoans (cit. [46]), indicating their evolution was parallel to insulin and its receptor. Caveolins contain a helix-turn-helix transmembrane domain [47] and may act as scaffolding proteins within caveolin-rich lipid rafts by compartmentalizing and concentrating signaling peptides bearing a caveolin-binding (CB) motif (reviewed in [45]). Using a glutathione-S-transferase (GST)-fusion protein containing the “caveolin-1 scaffolding domain” (residues 82–101) as a receptor to select peptide ligands from a bacteriophage display library, Couvet et al. [48] identified two related but distinct CB motifs, ΦxxxxΦxxΦ and ΦxΦxxxxΦ (where Φ is an aromatic amino acid and x any amino acid) in most of the proteins that have been shown to interact with caveolin.

**Table 5:** **Caveolin binding motifs**^**1 **^**present in the integral membrane proteins associated with insulin stimulation of the meiotic divisions in amphibian oocytes**

**Na**^**+**^**/K**^**+**^**-ATPase α-Subunit (Q92123)**	**Na**^**+**^**/Ca**^**2+ **^**exchanger (Q91849 )**	**Na**^**+**^**/H**^**+**^**-exchanger (P70009)**	**Na**^**+**^**/Glucose Co-transport (P31639)**	**Insulin receptor β-Subunit (Q9PVZ4)**
^87^WVKFCRQLFGGFSMLLW^103^	^367^FLTVFWKVF^375^	^6^FLNYCGYIF^14^	^526^YLYFAIVLF^534^	^451^WSFGVVLW^458^
TM-1 (100–122)	TM-1 (361–376)	TM-1 (6–26)	TM-13 (526–548)	
^986^WWFCAFPYSLLIFVY^999^	^371^FWKVFFAF^378^			^528^FQDVSFYY^536^
TM-10 (987–1003)	TM-1 (361–376)			TM-1(190–212)
	^496^FSVTLFTIF^504^			
	TM-5 (494–516)			

^1^Caveolin Binding Motif is defined as ФxxФxxxxФ or ФxФxxxxФ, where Ф is an aromatic amino acid (F, Y, W) and x is any amino acid. See ref. [46].

As shown in Table 5, two possible CB double motifs can be identified in the Na/K-ATPase α-subunit; one is present within TM-1, and a second within TM -10 [47]. TM -1 represents a double consensus sequence for a CB motif (^87^WVKFCRQLFGGFSMLLW^103^) to the motif, ΦxxΦxxxxΦxxΦxxxxΦ, whereas TM -10 displays the alternative double consensus sequence (^986^WWFCAFPYSLLIFVY^999^) to the motif, ΦxΦxxxxΦxxxxΦxΦ. For comparison, the Na^+^/Ca^2+^ exchanger contains 3 CB motifs, two overlapping TM-1 and a third overlapping TM-4. The Na^+^/H^+^-exchanger and SGLT2 contain one CB motif each, within TM-1 and TM-13, respectively. The last column of Table 5 indicates that the β-subunit of the insulin receptor (the putative membrane anchor - see Figure 6 for topology) contains 2 CB motifs (^451^WSFGVVLW^458^, ^528^FQDVSFYY^536^). However, neither CB motif is associated with the TM helix.

Although not shown here, the protease-activated receptor (PAR2) (Figure 6) contains three CB motifs: ^46^YIYTVAPF^54^, ^308^YSDSVYTFY^316^, ^333^YYFVSKDF^341^. The last two CB motifs overlap TM-7 and TM-8, respectively. The serine protease (O42272_XENLA) contains one CB motif (^313^FAALPFYW^320^) that coincides with the C-terminal TM helix. The amiloride-sensitive Na^+^-channel α-subunit (Accession #P51167) contains one CB motif: ^253^YSSFHHAIY^261^. The α and β subunits of the insulin receptor contain 1 and 2 CB motifs, respectively. The presence of CB motifs demonstrates that all nine integral membrane enzymes involved in insulin signaling are potential targets for interaction with caveolins.

### Role of Na^+^/K^+^-ATPase membrane pools in the meiotic divisions

Xie and Askari have proposed [49] that the α-subunit of Na/K-ATPase exists as two functional forms, each having a distinct but coupled function. One is the classical pool in which the enzyme acts as a energy transducing ion pump, and the second is associated with plasma membrane cell signaling systems. Liu and Askari subsequently found [50] that sealed isolated caveolae from myocytes exhibit active Na^+^ transport and that caveolar membranes contain about 75% of the total Na^+^/K^+^-ATPase activity. Studies by Liu et al. [51] demonstrated that the ouabain-sensitive Na^+^/K^+^-ATPase in the two pools have similar properties and equal molar activities, indicating that the caveolar enzyme retains its transport activity and does not contain nonpumping enzyme.

A variety of transport functions are known to depend on the maintenance of Na^+^ and K^+^ ionic gradients. The minimum functional unit of the Na^+^/K^+^-ATPase is a heteromeric dimer consisting of a catalytic α-subunit and a glycosylated β-subunits. Four isoforms of the α- subunit (each with a tissue-specific distribution) and three isoforms of the β-subunit have been identified [52]. An interesting finding has been that insulin induces translocation of α2 and β1 subunits from intracellular compartments to the plasma membrane in skeletal muscle [53], suggesting that insulin-induced subunit translocation may alter transport and/or signaling functions.

Based on: 1) the negative-going membrane hyperpolarization (Figure 4), 2) the 3-fold increase in high-affinity ouabain binding sites (Table 4), 3) the decrease in intracellular free Na^+^ (Table 2), and 4) the insulin-initiated membrane recycling, we suggest that insulin-induced Na^+^-pump activation may be due to recycling/recruitment of β and/or α-subunits to specific lipid rafts within the microregions of the plasma membrane. Na^+^-pump activation increases the Na^+^-free energy gradient, which could provide the energy source for the Na^+^:glucose symporter and the Na^+^:Ca^2+^ and Na^+^:H^+^ antiporters.

### Possible role of lipid rafts and caveolin-1 in receptor-ligand binding

Since all proteins involved (Na^+^/K^+^-ATPase, symporter, antiporters and the β-subunit of the insulin receptor) contain one or more CB motifs, it is likely that the insulin response system is localized to the caveolin-rich lipid rafts. Numerous studies have shown that plasma membrane compartmentalization occurs through lipid-lipid, lipid-protein and membrane-cytoskeletal interactions (reviewed in [54]). To date, two types of structures, planar rafts (also called “non-caveolar”) and the cholesterol-rich “lipid rafts” within caveolae (invaginations containing 21-24 kDa proteins called caveolins) have been described [54]. Lipid rafts are more ordered and tightly packed than the surrounding bilayer; both are estimated to be 25–100 nm in diameter. Liu and Askari report [49] that caveolar membranes from cardiac myocytes contain about 75% of total myocyte Na/K-ATPase activity and that sealed, isolated caveolae exhibit active Na^+^ transport. These authors suggest that the caveolar impocketings are the “primary portals for Na^+^ and K^+^ fluxes” [49], and represent sites where the ion pumping and signaling functions of Na/K-ATPase are integrated.

Sequence analysis of the insulin receptor β-subunit, membrane serine protease, PAR2 and the amiloride-sensitive Na^+^ channel subunit α (Accession #P51167) (Figure 6), as well as the Na^+^/K^+^-ATPase α-subunit, Na^+^:glucose symporter, Na^+^/Ca^2+^ and Na^+^/H^+^ antiporters (Figure 7), indicate that all contain caveolin binding (CB) motifs. This implicates the low-density (caveolar) membrane domains in insulin-mediated signaling associated with prophase block, and suggests that the insulin-induced shift from low affinity to high affinity ouabain binding sites (Table 4) results from an increase in caveolar (lipid raft) Na^+^-pump sites during the initial transient increase in membrane recycling (Figure 3). A role for lipid rafts in meiosis is further supported by the finding by Sadler and Jacobs [55] that treatment of *Xenopus* oocytes with the cholesterol-depleting drug, methyl-β-cyclodextrin, removed 50–70% of cell-associated ^3^H]cholesterol and accelerated the progesterone response. We find that progesterone induces net internalization of 40–50% of the oocyte surface containing >95% of the Na^+^/K^+^-ATPase α-subunit just prior to nuclear membrane breakdown [8], consistent with a loss of membrane caveolin-rich rafts.

## Conclusions

The data presented here indicate that, under physiological conditions, resumption of meiotic divisions and continuation to second meiotic metaphase arrest requires the action of both insulin and progesterone at caveolar surface receptors. The insulin-induced membrane changes are potentiated by the non-genomic action of progesterone released by follicle cells in response to gonadotropin (reviewed in [5]). As noted in Background, insulin evolved very early and occurs in at least two main branches of the phylogenetic tree. Studies on the evolutionary pathway of the insulin gene family suggest that insulin and insulin-like growth factor (IGF) became distinct molecules only after vertebrates appeared (cit. [56]). McRory and Sherwood [56] conclude that insulin and IGF maintained separate gene lineages in both vertebrate and prochordate evolution and have a distinct evolutionary history of at least 600 million years. Steroids, on the other hand, evolved as the atmospheric oxygen content rose to a level sufficient to sustain oxygenase reactions [57]. The initial pathway leading to steroid synthesis depends upon anaerobic formation of acetyl-CoA via mevalonate and farnesyl pyrophosphate to form squalene. An oxygenase then converts squalene to epoxysqualene using molecular oxygen. Molecular oxygen is required for a number of the 19 reactions required for processing the cyclization products that lead to cholesterol formation.

Current consensus indicates that measurable amounts (0.5%) of oxygen first appeared in Earth’s atmosphere some 2.4 billion years ago, with a second large increase to about 20% in atmospheric oxygen occurring around 600 million years ago [58]. Primitive vertebrates first appeared about 525 million years ago, following a second and much larger increase in atmospheric oxygen. Thus, insulin-like peptides evolved about 2 billion years ago, followed by the much later appearance of a non-genomic progesterone action coupled to one or more insulin-response systems. The non-genomic action of progesterone (the first true steroid) associated with meiosis thus evolved as additional modulator(s) of existing peptide signaling systems and may have preceded the genomic action of steroids at the cell nucleus.

Taken *in toto*, the evidence outlined here suggests that insulin initiates a transient increase in plasma membrane turnover (endocytosis) within the first 5–10 min, resulting in decreased Na^+^ conductance (Table 2), followed by a shift from the low-affinity to the high-affinity ouabain binding (pumping) forms of Na/K-ATPase (Table 4). The fact that both the α and β-subunits of the insulin receptor as well as the response system enzymes (Table 5) contain one or more caveolin binding motifs, indicates that the insulin response system is located in caveolar lipid rafts. In adipocytes the insulin receptor has been localized to plasma membrane caveolae, and is internalized within minutes upon addition of insulin [59]. Thus, internalization and recycling could account for the selective increase in high affinity ouabain binding sites in the oocyte plasma membrane and could initiate protease activation (Figure 5) with the concomitant loss of Na^+^ channels (Figure 3).

Following a rise in circulating insulin as females come out of hibernation in late spring, oocyte membrane hyperpolarization resulting from decreased Na^+^ conductance is the principal driving force for the increase in 2Na^+^/glucose uptake that provides an energy source for meiosis and early cleavage. The increased Na^+^ free energy gradient, in turn, provides the energy for the enhanced 3Na^+^/Ca^2+^ and Na^+^/H^+^ antiporters, resulting in increased Ca^2+^ efflux and alkalinization of the oocyte cytosol. As indicated by the topology (e.g., transmembrane helices, caveolin binding motifs) of the enzyme systems involved (Figures 6 and 7 and Table 5), insulin binding to lipid raft sites may initiate a complex sequence of inter- and intramolecular helix-helix interactions to choreograph the sequential changes in plasma membrane ion permeability, transmembrane potential and intracellular pH essential for the meiotic divisions.

## Methods

### Materials

Gravid *Rana pipiens* females from the northeastern United States were purchased from Connecticut Valley Biologicals, Southampton, MA, and maintained in hibernation at 5–7°C. Steroids were obtained from Steraloids Inc. (Newport, RI). Sodium insulin was obtained from Eli Lilly and Company and dissolved in Ringer’s solution immediately before use. [^3^H]Ouabain (22.6 Ci/mmol), [^14^C]inulin, [^125^I]insulin (100 Ci/μg), 2-Deoxy-D-[U-^14^C]glucose (0.6 mCi/mmol), ^45^Ca (final Sp. Act. 40–50 mCi/mmol) and carrier-free ^22^Na were obtained from New England Nuclear, Boston, MA. Modified Ringer’s solution contained 111 NaCl, 1.9 mM KCl, 1.1 mM CaCl_2_, 0.8 mM MgSO_4_, 2.3 mM NaHCO_3_, and 0.08 mM NaHPO_4_. The ionic composition of this amphibian Ringer’s solution is based on that of frog plasma and differs from “Barth’s” or various “OR-2” solutions used in *Xenopus laevis* experiments. Progesterone and ouabain were dissolved in 95% ethanol; 1.0 μl was added per ml of Ringer’s solution with shaking for 30 sec, followed by 1:10 serial dilutions. The final ethanol content was 0.01%.

### Use of *Rana pipiens* oocytes

The *R. pipiens* oocyte is particularly appropriate for studies of steroid or insulin action at the plasma membrane. Unlike *X. laevis* ovaries, which contain several stages of growing oocytes (only “banded” oocytes respond to gonadotropin) [60], the mature *R. pipiens* ovary contains 1–3 thousand fully grown oocytes in meiotic prophase arrest, all of which undergo synchronous meiotic divisions in response to progesterone. *R. pipiens* oocytes grow and store yolk during the summer prior to the frog’s entry into hibernation in the late fall. Oocytes and/or follicles were obtained from hibernating gravid females from January through April. Each oocyte is a giant cell, 2.0–2.3 mm in diameter. Intact plasma-vitelline membranes can be isolated and used to study ligand binding (e.g. [5]). Several hundred oocytes collected from a single female are sufficient to characterize ligand binding and the sequential changes in plasma membrane potential and surface area (capacitance). Nuclear membrane breakdown or appearance of the first polar body can be measured in denuded oocytes under a low power binocular microscope. Oocytes are heat-fixed for 5 min, opened with fine-tipped watchmakers’ forceps, and the intact nucleus can be seen as a large (0.5 mm) sphere and becomes a diffuse whitish region in the animal hemisphere after breakdown. Schultz in 1887 was the first to describe the emergence of the first polar body from the egg of the frog. He stated (in German) that it was “darkly pigmented, and emerged from a small pit (fovea) generally toward the center of the animal hemisphere” (reviewed in [15]).

### Secondary structure and transmembrane helix predictions

Protein secondary structures were predicted using PSIPRED v. 3.0 [61]. TMHMM projections used in Figures 6 and 7 were generated using the server at the Center for Bological Sequence Analysis, Technical University of Denmark DTU. Positions of transmembrane helices were compared using: 1) the TOPCONS algorithm [62] (http://topcons.cbr.su.se/), 2) the ConPred II server [63] (http://www.hsls.pitt.edu/obrc/index.php?page=URL1098386137), 3) SOSUI ver. 1.1 [64] (http://bp.nuap.nagoya-u.ac.jp/sosui/) and 4) Phobius [65]. SignalP v. 4.0 [44] servers were used to predict signal peptide locations.

### [^3^H] Ouabain and [^125^I] insulin binding studies

In each experiment, 6–12 denuded oocytes were incubated in a minimum volume of 2.0 ml Ringer’s solution containing a specific concentration of ^3^H]ouabain (22.6 Ci/mmol) or ^125^I]insulin (100 Ci/μg) as indicated. Oocytes were kept slowing moving on a rotating table and were handled using glass pipettes with a bore diameter slightly larger than the oocyte. Ligand concentrations did not become rate limiting at the various times and concentrations used. At the times indicated, six oocytes were removed, rinsed five-times with fresh Ringer’s solution, and transferred to a buffered sucrose solution and the plasma-vitelline membrane complex isolated as described elsewhere [5].

### Na^+^ and Ca^2+^ efflux and pH_i_ measurements

^22^Na^+^ and ^45^Ca^2+^ uptake and exchange has been described previously [6]. For efflux studies, denuded oocytes were preloaded with ^22^Na^+^ (final Sp. Act 40–50 Ci/mmol) for 2 h at 20°C, and then rinsed with Ringer’s solution for two min and 0.5 ml fractions collected using a laminar- flow method. Individual experiments were carried out with groups of 6–8 oocytes.

Intracellular pH measurements using ^31^P-NMR required relatively larger samples (200–250 oocytes) so that isolated follicles were used. However, as noted previously, similar pH_i_ values (±0.03 pH units) were obtained when denuded oocytes and follicles were compared [25].

Follicles were loosely packed in a 10 mm diameter tube and superfused with aerated Ringer’s solution at 20–21°C (see [8]). ^31^P-NMR spectra were recorded at 81 MHz with a Varian VXR-200 and pH_i_ measured as described previously [25].

### Oocyte membrane potential and capacitance measurements

Membrane potential measurements were made using a W-P Instruments (New Haven, CT) M-707 microprobe electrometer and standard 2.5 M KCl-filled glass microelectrodes (5 to 15 MΩ DC resistance). The denuded oocytes were kept in a bath connected to ground via a 2.5 M KCl-agar bridge and a Ag-AgCl electrode. Electrodes were inserted normal to the surface of the animal hemisphere, approximately 45° from the animal pole. Electrode tip penetration was usually 100–200 μm into the oocyte cytoplasm. Oocytes were voltage-clamped by feeding the oocyte potential signal into a negative feedback circuit, the output of which was fed back into the oocyte by a second microelectrode. Clamping current was measured by recording the voltage drop across a precision resistor placed in series with the feedback micropipette.

### Na^+^ activity measurements

The Na^+^-sensitive microelectrode consisted of an outer tapering sheath of pyrex capillary tubing with a protruding Na^+^-sensitive tip of Corning NAS 11–18 capillary tubing. The Na^+^-sensitive tip was about 2–4 μm in diameter and extended about 200 μm beyond the outer insulating tip. The Na^+^-sensitive tip of the inner micropipette and the junction between the Na^+^-sensitive glass and insulating sheath were heat-sealed. Measurements were made on denuded oocytes and isolated follicles as described previously [66].

### Calculation of glucose and Na^+^ free energy gradients

The movement of any molecule or ion down, or up, a concentration gradient involves a change in free energy, ΔG; Down releases energy so ΔG is negative; Up consumes energy so ΔG is positive. The amount of free energy released or consumed can be calculated from the equation:

(1) $$\Delta G=\text{nRTln}\left\{{\left[X\right]}_{i}/{\left[X\right]}_{o}\right\}+{\text{nzFV}}_{m}$$

where ΔG is the change in free energy, n is the # of moles of ions/nutrients moved across the membrane; R = 2, the “gas constant”(units are cal/mole/°K); T = °K, the absolute temperature =°C + 273; z = the charge on the ion/glucose (+1 for Na^+^, 0 for glucose); F = 23,062, the calories released as one mole of charge moves down a voltage gradient of 1 volt (1000 mV); V_m_ = the membrane potential, about − 60 mV in insulin treated amphibian oocytes; [X]_i_ and [X]_o_ are intracellular and extracellular concentrations, respectively. The driving force for the active transport of glucose (and other small organic molecules, e.g., amino acids) uphill against their concentration gradient is the force provided by the movement of sodium ions down their electrochemical gradient. It should be noted that ultimately, the driving force is provided by the energy of ATP synthesized in cellular respiration. This is because the sodium gradient across the plasma membrane is created by the active transport of Na^+^ out of the cell by the Na^+^/K^+^ ATPase, the single most profligate user of energy in most living cells.

In the case of glucose, insulin usually stimulates the co-transport of 2 Na^+^ ions for each glucose molecule. The ability of SGLT’s to accumulate glucose is critically dependent on the stoichiometry of the Na^+^ and glucose fluxes, and yields:

(2) $${\left[\text{Glucose}\right]}_{i}/{\left[\text{Glucose}\right]}_{o}={\left({\left[{\text{Na}}^{+}\right]}_{o}/{\left[{\text{Na}}^{+}\right]}_{i}\right)}^{2}/\text{exp}\left({2V}_{m}F/\text{RT}\right)$$

where i and o are intracellular and extracellular glucose and Na^+^ concentrations, respectively.

### Analysis of plasma membrane protease activity

Protease activity was determined by monitoring changes in absorbance during hydrolysis of an amino acid ester (α-p-Toluenesulfonyl-L-arginine methyl ester hydrochloride TAME HCl) with a Gilford spectrophotometer, using a modification of the method of Hummel [67]. The assay mixture contained 1.0 mM TAME HCl, 0.24 M sucrose, 1.1 mM CaCl_2_, and 0.2 M tris(hydroxymethyl)aminomethane at pH 8.1. Aliquots of Ringer’s solution, oocyte cytosol, or 5–10 intact plasma-vitelline membranes were suspended in 0.9 ml of the assay mixture, in a quartz cell with a 1 cm light path. The reaction was initiated by addition of 0.1 ml insulin prepared in the same assay mixture and the contents were agitated for 5–10 s with a small teflon “plumper”. Absorbance was measured at 247 μm (20–22°C) at 30 second intervals and the difference spectra calculated with respect to the control samples. 0.2 M phenylmethanesulfonyl fluoride (PMSF) was dissolved in 2-propranol immediately before use and μl aliquots added to the assay medium.

### Use of animals

The frogs used in this study were handled according to the animal use protocol reviewed and approved by the Institutional Animal Care and Use Committee (IACUC) of the Albert Einstein College of Medicine. The Einstein IACUC is responsible for welfare, care and treatment of research animals, and ensures the most humane care possible and compliance with all animal welfare and health and safety policies and regulations of the USDA and PHS. Health care of experimental animals is provided by Institute of Animal Studies (IAS) veterinarians and veterinary technicians. The IAS also coordinates the purchase of experimental animals and provides animal husbandry services.

## Competing interest

The authors declare that they have no competing interests.

## Authors’ contributions

The approach was conceived of by all four authors and the experiments were carried out by GAM, ABK and RKG. GAM wrote the draft of the manuscript and it was edited by all authors. All authors read and approved the final manuscript.
